# Gut Microbiome Diagnostic Biomarkers for Colorectal Cancer

**DOI:** 10.5152/tjg.2025.24810

**Published:** 2025-09-22

**Authors:** Fei Shen, Chenzhou Xu, Chao Wang

**Affiliations:** Department of Gastroenterology, The First Hospital of Jiaxing, Affiliated Hospital of Jiaxing University, Jiaxing, China

**Keywords:** Colorectal cancer, diagnosis, gut microbiota, microbial markers, precancerous lesions, treatment monitoring

## Abstract

**Background/Aims:**

Gold standard diagnostic methods, such as invasive procedures and serum biomarkers, have limited sensitivity and specificity for the detection of colorectal cancer (CRC). Thus, the development of more accurate and noninvasive detection approaches is imperative. Emerging research elucidating the intricate role of the gut microbiota in CRC pathogenesis underscores the need for precision screening tailored to high-risk cohorts to improve early detection and intervention strategies and comprehensively address this challenging clinical problem.

**Materials and Methods:**

Fecal metagenomic sequencing datasets were employed to identify potential bacterial biomarkers for CRC diagnosis and selected relevant microbial taxa for subsequent validation. A total of 180 participants were enrolled: 65 healthy controls (HC), 65 colorectal adenoma patients, and 50 CRC patients, and fecal samples were analyzed using fluorescence quantitative polymerase chain reaction to confirm biomarker relative abundance, culminating in the establishment of an evolutionary model for CRC progression; furthermore, a treatment efficacy and prognostication model supported by comprehensive statistical methodologies was established.

**Results:**

This study analyzed fecal microbial biomarkers associated with CRC progression and identified differentially abundant bacterial species across HCs, adenoma, and CRC patient groups. Notably, *Fusobacterium nucleatum* (*Fn*) and *Peptostreptococcus anaerobius* (*P*. *anaerobius*) showed significant correlations with CRC stage and metastasis, highlighting their potential as diagnostic biomarkers. Among individual microbes, *P*. *anaerobius* exhibited the highest diagnostic value when combined with *Fn*.

**Conclusion:**

The results underscore the potential application of fecal microbial markers, particularly *Fn* and *P*. *anaerobius*, for diagnosing CRC and monitoring its progression.

**Main Points**

- Gut microbiota alterations, particularly the abundance of *Fusobacterium nucleatum* (*Fn*), *Peptostreptococcus anaerobius* (*P*. *anaerobius)*, and enterotoxigenic *Bacteroides fragilis*, correlate with colorectal cancer (CRC) progression and metastasis.
- Fecal microbiota biomarkers, including *Fn* and *P*. *anaerobius*, showed strong diagnostic value, with area under the receiver operating characteristic curves of 0.822 and 0.830, respectively, for distinguishing CRC from healthy controls.
- *Fusobacterium nucleatum* significantly enhanced CRC cell migration and metastasis in both in vitro and in vivo models, suggesting its role in promoting CRC progression.
- The study demonstrated a progressive increase in the abundance of certain gut bacteria from healthy individuals to adenoma and CRC patients, supporting their potential as early biomarkers for CRC detection.
- The combination of microbiota markers, such as *Fn* and *P*. *anaerobius*, could improve diagnostic sensitivity for CRC, potentially augmenting existing screening methods like Fecal occult blood test (FOBT).

## Introduction

Colorectal cancer (CRC) has a high incidence and is the second most common cause of mortality.^1^^,^^2^ The incidence rates of CRC were observed to be higher in younger adults (age <50 years), and most cancer patients are diagnosed in the middle or late stages, where treatment effectiveness and prognosis are poor, making early diagnosis and treatment crucial.^3^ To date, the quest to solve the pivotal challenge of early detection and intervention in CRC remains the main goal due to the objectivity and inaccuracy of current methods.^4^ Additionally, several noninvasive serum biomarkers, such as carcinoembryonic antigen (CEA), carbohydrate antigen 199 (CA199), and carbohydrate antigen 50 (CA50), have been integrated into clinical practice.^5^ However, these markers have limited clinical utility owing to their subpar sensitivity and specificity.^6^^-^^8^ Moreover, microRNAs, long noncoding RNAs (lncRNAs), and long noncoding RNAs (circRNAs) have been implicated in the prognostication and detection of CRC.^9^^-^^11^ However, these markers largely remain in the research phase without established systemic diagnostic models or robust clinical correlation analyses. Thus, there is an exigent need to develop sensitive, noninvasive, and economically viable systems for CRC detection and prognostic monitoring.

## Materials and Methods

## Validation of Efficacious Microbiota Biomarkers for Colorectal Cancer

### Cohort Description and Stratification Norms:

In this investigation, a total of 180 participants were enrolled, including 65 healthy controls (HC), 65 colorectal adenoma (CRA) patients, and 50 CRC patients. Fecal specimens from the CRC cohort were systematically collected preoperatively. Informed consent was obtained from all participants. Participants were recruited from the hospital between 2024 and 2025. The study included individuals who underwent routine health examinations or colorectal screenings. All subjects provided informed consent, and the Medical Ethics Committee of The First Hospital of Jiaxing approved this study (2024-KY-009) according to the World Medical Association Declaration of Helsinki on January 9, 2024.

The inclusion criteria were as follows: (i) aged 40-75 years; (ii) confirmed CRC diagnosis by colonoscopy and pathological examination; (iii) body mass index (BMI) 18.5-30 kg/m^2^; and (iv) the absence of distant metastases or curative resection; (V) All of the patients whom were previously selected expressed the 4 major MMR proteins (MLH1, MSH2, MSH6, and PMS2), only patients with proficient MMR (pMMR) status were included, while those with MMR deficiency (dMMR) were excluded.

The exclusion criteria were as follows: (i) age >75 years; (ii) BMI >30 kg/m^2^; (iii) pregnancy; (iv) the presence of other tumors; (v) the presence of mental illness; (vi) received any antibiotic treatment within 3 months prior to sample collection; (vii) history of gastrointestinal surgery; (viii) participation in other related experimental drug trials within 2 months before sample collection; (ix) inability to provide informed consent; and (x) had a fecal occult blood test or used related medications within 6 months before sample collection.

### Database Selection

Four publicly available fecal metagenomic datasets from France, China, Austria, and the USA were downloaded and processed uniformly, retaining only bacterial sequences for analysis and excluding any reads from viruses, archaea, or eukaryotes. Bacterial taxa shared across all 4 cohorts were then identified, and each was evaluated for its discriminatory power in distinguishing HCs, colorectal adenoma, and CRC samples. Five candidate microbial biomarkers were thus selected*: Fn, P*.*anaerobius, enterotoxigenic Bacteroides fragilis, Bifidobacterium, *and* Lactobacillus*. These candidates were subsequently validated by quantitative polymerase chain reaction (qPCR) in a prospective cohort of 180 local participants. This multi-stage approach was designed to identify robust fecal microbial biomarkers for early CRC diagnosis.

### Collection and Analysis of Samples

After providing informed consent, fecal specimens were collected 1-3 days before the intestinal preparatory phase. Genomic DNA from the fecal samples was extracted utilizing the TIANamp Stool DNA Kit according to the manufacturer’s protocol, and the resulting fecal microbiota genomic DNA was preserved at −80°C. To confirm the identified microbial biomarkers, fluorescence qPCR was employed to assess the relative abundance of distinctive bacterial taxa across groups. The relative expression of fecal bacterial DNA was compared to an internal reference and quantified utilizing the delta Ct (∆Ct) method inherent to qPCR.

## Establishment of the “Precancerous Lesions-Colorectal Cancer” Evolution Model

Receiver operating characteristic (ROC) curves were generated to distinguish between normal controls, patients with precancerous colorectal lesions, and patients with CRC. This study included healthy individuals, those with precancerous lesions, and patients with CRC. These patients were subjected to triannual, biannual, and annual assessments of microbial markers, along with annual colonoscopic examinations for follow-up. By integrating and comparing patient pathology, diagnostic results, endoscopic images, laboratory tests, and other clinical case data, a microbial model for early warning of the evolution from “precancerous lesions to colorectal cancer” was established.

## Establishment of a Colorectal Cancer Treatment Efficacy and Prognostication Model

In the cohort of CRC patients, fecal samples were collected before and after treatment; the patients included those receiving postoperative care, adjunct chemotherapy, or exclusive chemotherapy regimens. These samples were subjected to fluorescence quantitative PCR to quantify microbial biomarkers, thereby tracking shifts in the fecal microbiota that were indicative of disease trajectory. The patient follow-up protocol included triannual assessments of microbial biomarkers and annual diagnostic evaluations via colonoscopy or radiologic imaging. A comprehensive analysis involving imaging outcomes, endoscopic visuals, laboratory diagnostics, extensive clinical records, patient medical history, and a spectrum of bioinformatics data underpinned the construction of a microbiota-based framework. This framework was dedicated to monitoring therapeutic outcomes in patients with CRC and forecasting potential recurrence.

## Cell Culture and Reagents and Quantitative Real-Time Polymerase Chain Reaction

The human colorectal carcinoma cells (HCT-116) and LoVo were obtained from the American Type Culture Collection (ATCC). All cells were cultured in DMEM/HIGH GLUCOSE medium (Hyclone, USA) containing 10% fetal bovine serum (Sijiqing, China) at 37°C in 5% CO_2_ incubator. Genomic DNA was extracted from fecal samples using the TIANamp Stool DNA Kit (TIANGEN, China) according to the manufacturer’s protocol. The abundance of target bacterial species was quantified by real-time PCR using species-specific primers. Quantitative real-time PCR (qRT-PCR) was performed on the Lightcycler480II (Roche, Switzerland). *Fusobacte rium nucleatum* strain ATCC 25586 was obtained from the ATCC. The bacteria were cultured anaerobically in Columbia blood agar or brain heart infusion broth supplemented with 5% defibrinated sheep blood at 37°C. All bacterial cultures were handled under strict anaerobic conditions using an anaerobic chamber (Table 1).

### Statistical Methodology

Statistical analyses were conducted using GraphPad Prism 7 (GraphPad Software Inc.; San Diego, CA, USA) and SPSS 20.0 (IBM SPSS Corp.; Armonk, NY, USA). For datasets conforming to normal distribution and homogeneity of variance, Student’s t test and one-way ANOVA were used to assess intergroup differences. In cases of non-normal distribution or variance heterogeneity, nonparametric tests such as the Mann–Whitney *U* test and Kruskal–Wallis test were applied. Categorical variables were analyzed using Pearson’s chi-squared (*χ*^2^) test.

Normalization of qPCR data was performed using the ∆Ct method, with gene relative abundance calculated relative to an internal reference gene. All qPCR reactions were conducted in triplicate, and mean ∆Ct values were used for comparative analyses. To control for potential confounders such as age, sex, and BMI, these variables were assessed across groups, showing no significant differences (*P* > .05). In addition, multivariate regression models were used to adjust for these covariates in diagnostic evaluations. The diagnostic performance of microbial markers was evaluated by computing the area under the receiver operating characteristic curve (AUC). To correct for multiple comparisons, the Benjamini–Hochberg false discovery rate method was applied where appropriate. A two-tailed *P* value < .05 was considered statistically significant.

## Results

## Identification of Differentially Expressed Microbial Biomarkers in Colorectal Cancer

In the initial phase of this study, comprehensive searches were conducted across multiple publicly available databases, resulting in the identification and inclusion of 228 samples, comprising 89 HC samples, 89 colorectal adenoma samples, and 225 CRC fecal samples, all of which were subjected to macro-genomic sequencing (Table 2). After amalgamating the data from these 4 datasets, a comparative analysis was performed among the HC group, the adenoma group, and the CRC group, revealing differential abundances of 24 distinct bacterial species. Specifically, 8 microbial markers were detected in the HC group, 3 in the adenoma group, and 13 in the CRC group (Table 3).

Notably, the fecal microbial composition in the HC group predominantly featured markers from the Lactobacillus and Bifidobacterium genera, whereas the CRC group exhibited a prevalence of markers such as *Fn*, *P*.*anaerobius*, and enterotoxigenic* Bacteroides fragilis*. These 5 disparate microbial entities were selected as the preliminary pool of microbial markers for CRC and subjected to subsequent in-depth validation.

## Assessment of Characteristic Gut Microbial Marker Expression in 3 Distinct Groups: the Normal Group, the Colorectal Adenoma Group, and the Colorectal Cancer Group

Following a stringent selection process adhering to the inclusion and exclusion criteria established in this study, a total of 50 patients who were diagnosed with CRC (in the CRC group) were successfully enrolled from the local area, along with 65 patients who presented with colorectal adenomas (in the CRA group), 25 individuals with advanced adenomas (in the AA group), 40 subjects with non-advanced adenomas (in the non-AA group), and an additional 65 HCs (in the HC group), as outlined in Table 4. The age, gender, and BMI distributions appear comparable across groups, with no statistically significant differences (*P* > .05) between the HC, CRA, and CRC groups.

**Figure 1.**
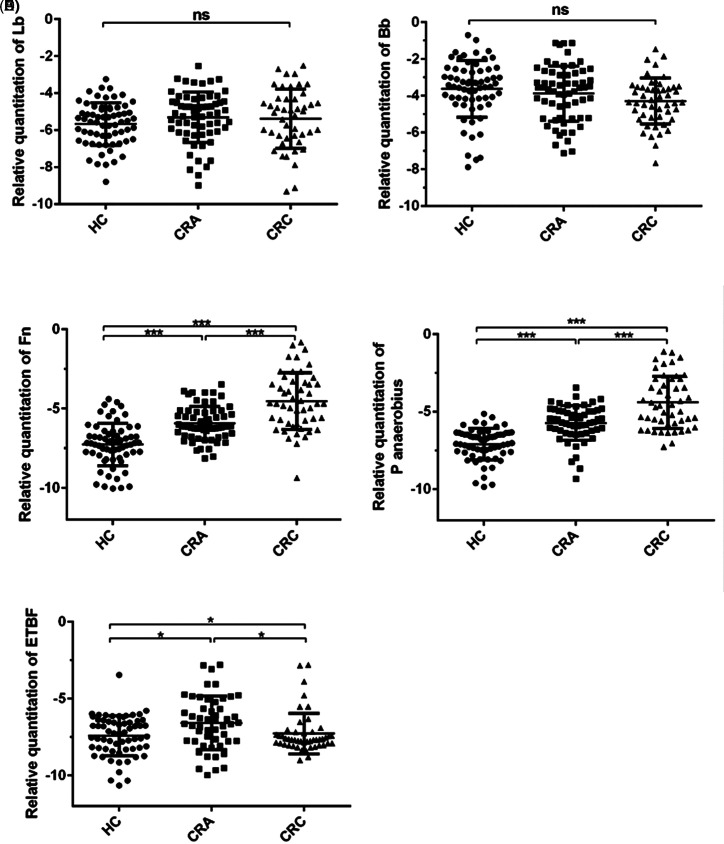
Comparison of characteristic fecal microbial marker levels among different groups. The relative levels of fecal bacteria in the colorectal cancer, colorectal adenoma, and healthy control groups are shown. *Lactobacillus genus* (Lb) (A), *Bifidobacterium genus* (Bb) (B), *Fusobacterium nucleatum* (*Fn*) (C), *Peptostreptococcus anaerobius* (*P*. *anaerobius*) (D), and enterotoxigenic *Bacteroides fragilis* (ETBF) (E). Note: * indicates *P* < .05, ** indicates *P* < .01, *** indicates *P* < .001, ns indicates no statistical significance.

The findings revealed a progressive increase in the relative levels of *Fn* and *P*.*anaerobius* in direct correlation with the transition from a “healthy” state to “adenoma” and further to “cancer” (*P* < .001). Notably, the relative abundance of enterotoxigenic *Bacteroides fragilis* (ETBF*)* in the CRA group surpassed that in both the HC group and the CRC group (*P* < .05) (Figure 1E). The cutoff values for each microbial marker were determined using the Youden Index (J = sensitivity + specificity − 1), which identifies the threshold that optimally balances sensitivity and specificity on the ROC curve. Based on visual estimation from Figure 1, the approximate cutoff values were 2.3 for *Fn* and 2.1 for *P*. *anaerobius* (relative abundance units). These thresholds effectively distinguished CRC and CRA patients from HCs. Conversely, the abundances of Lactobacillus and Bifidobacterium (Figure 1A and B) were not significantly different among the HC, CRA, and CRC groups (*P* > .05).

## Relative Abundance of Gut Microbial Markers in Different Stages of Colorectal Adenoma and Colorectal Cancer

**Figure 2.**
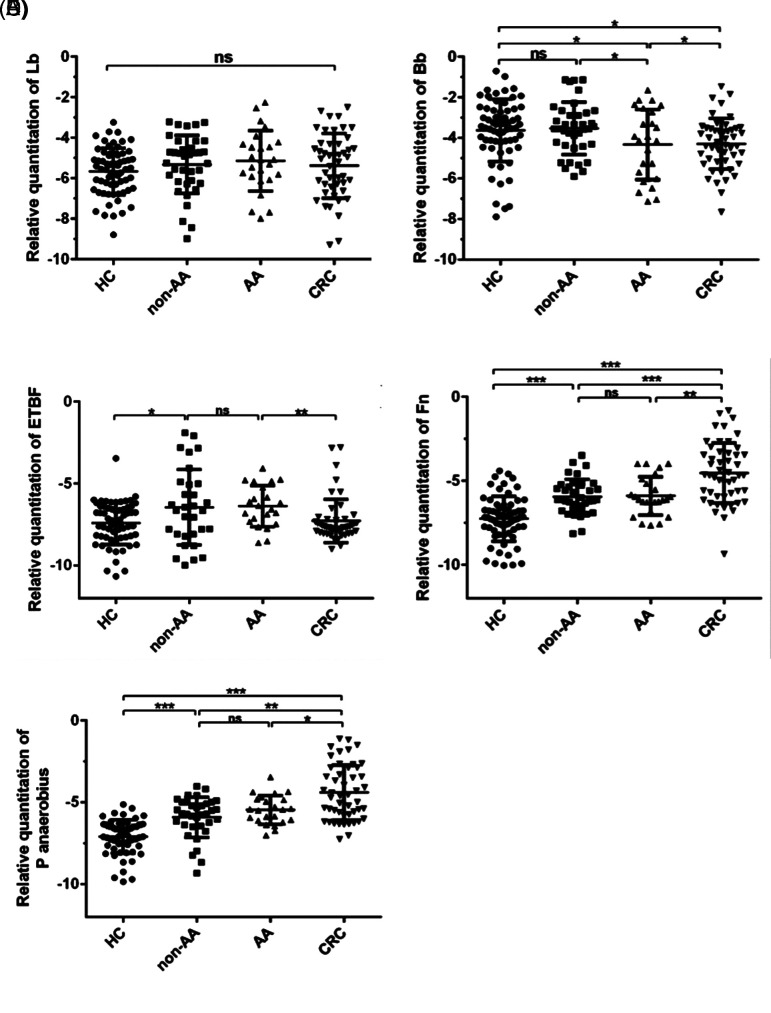
Comparison of gut microbial marker levels in patients in different stages of adenoma and colorectal cancer. The relative quantity of Bb was lower in the advanced adenoma group than in the non-advanced adenoma group (B). However, there were no significant differences in the relative abundance of the remaining 4 bacteria (Lb, ETBF, *Fn*, and *P*. *anaerobius*) among the adenoma groups at different stages (A).

To conduct an objective analysis of the interplay among the selected gut microbial markers throughout the progression from colorectal adenoma to cancer, the 2018 Colorectal Diagnosis and Treatment Guidelines, as revised by the Chinese Society of Clinical Oncology, were employed as a reference. Subsequently, the colorectal adenoma cohort was categorized into 2 distinct subgroups: the non-advanced adenoma group and the advanced adenoma group. A comparative assessment was then conducted with the CRC group. As depicted in Figure 2, these findings indicate a notable reduction in the relative abundance of the genus Bifidobacterium within the advanced adenoma group in comparison to the non-advanced adenoma group (Figure 2B). In contrast, the relative abundance of the remaining 4 microbial markers exhibited no statistically significant disparities among the adenoma subgroups at varying stages.

## Correlation Analysis of Gut Microbial Markers and the Clinical Stage of Colorectal Cancer

**Figure 3.**
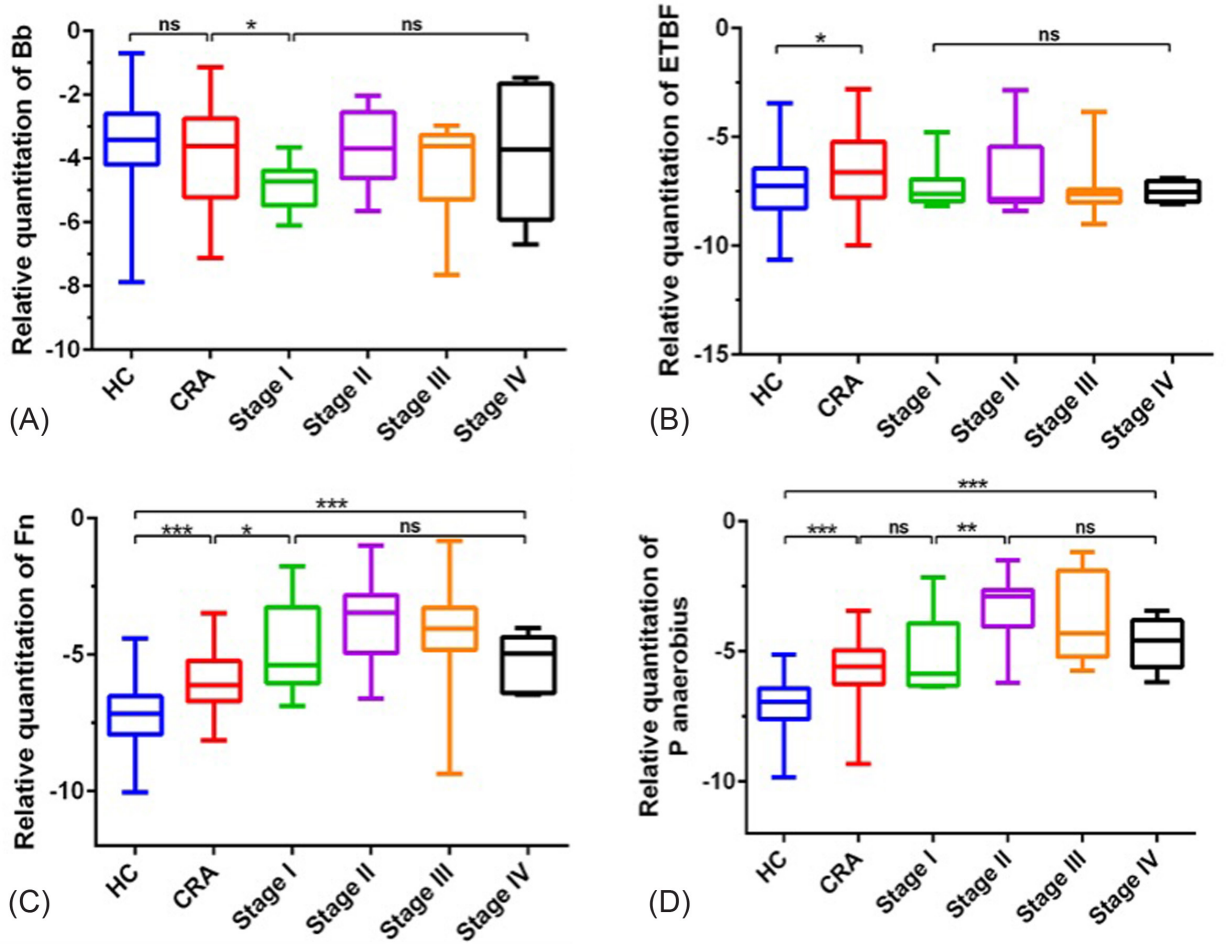
Relative quantitative comparison of gut microbial markers in patients in different TNM stages. The relative levels of Bifidobacterium, *Fn*, and *P*. *anaerobius* did not significantly differ among the patients in different TNM stages (*P* > .05) (A, B, and C). However, the relative quantity of anaerobic Streptococcus was significantly greater in patients with stage II-IV disease than in patients with stage I disease (*P* < .01) (D).

To conduct an in-depth analysis of the correlation between the selected gut microbial markers and the various clinical stages of CRC, the staging criteria outlined in the 8th edition of the American Joint Committee on Cancer (AJCC)/International Union Against Cancer (UICC) Tumor Nodes Metastasis (TNM) staging system was meticulously followed. The study cohort comprised 19 patients with stage I CRC, 10 patients with stage II CRC, 15 patients with stage III CRC, and 6 patients with stage IV CRC. The aim was to scrutinize whether there were any discernible trends or alterations in the levels of these 5 gut microbial markers across the different TNM stages of patients with CRC. As illustrated in Figure 3, the analysis revealed that the relative levels of the Bifidobacterium genus, *enterotoxigenic Bacteroides fragilis*, and *Fn* exhibited no statistically significant variations across diverse TNM stages (*P* > .05) (Figure 3A-C). However, notably, the relative quantity of *P*.*anaerobius* was substantially greater in Stages II to IV than in Stage I (*P* < .01) (Figure 3D).

## In Vivo and In Vitro Experiments Demonstrated That *Fusobacterium nucleatum* Promotes Colorectal Cancer Metastasis

**Figure 4.**
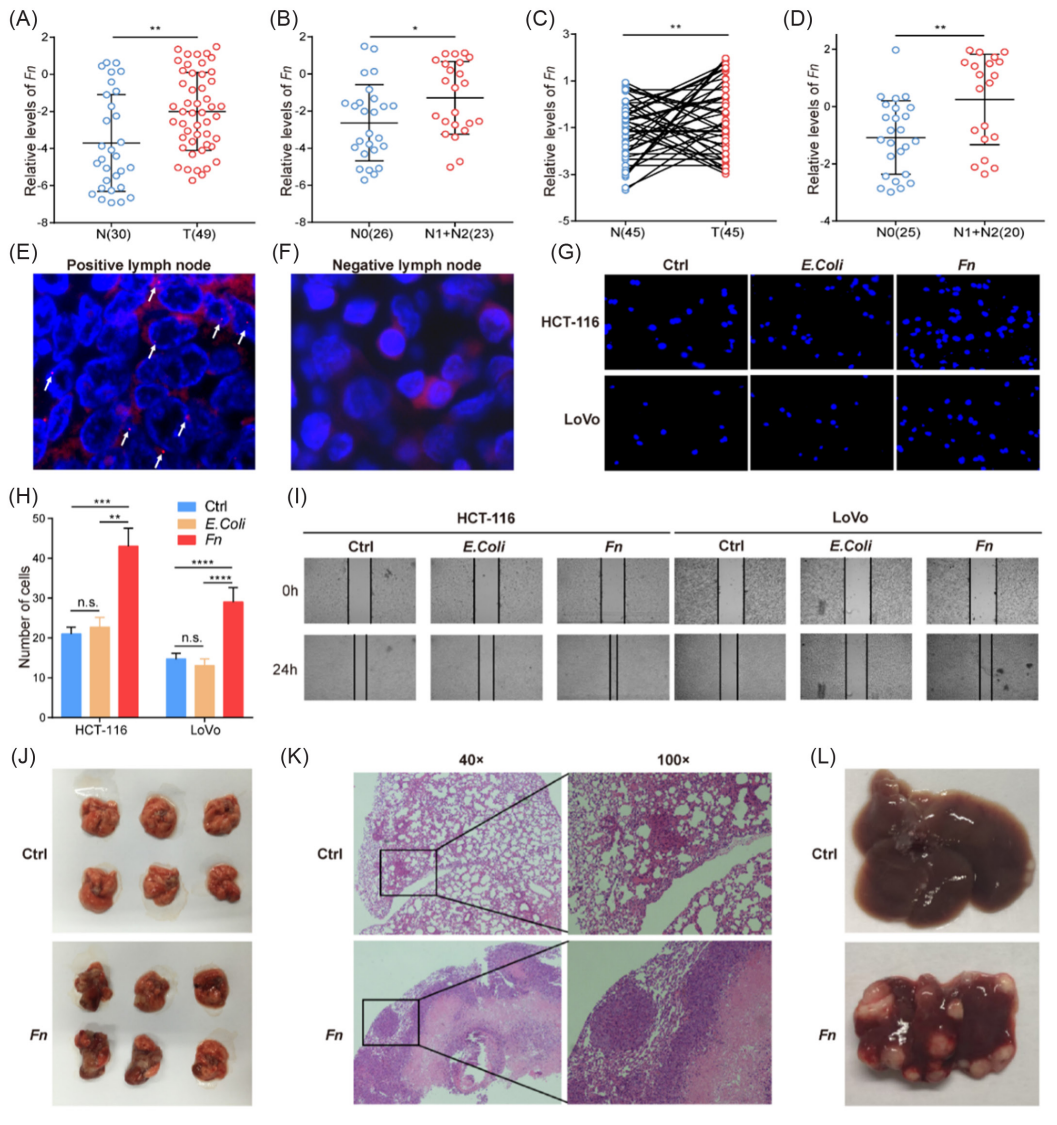
*Fusobacte rium nucleatum *(*Fn*) promotes colorectal cancer metastasis. Polymerase chain reaction analysis revealed an increased abundance of *Fn* in the feces of patients with colorectal cancer, with a significantly greater abundance in patients with lymph node metastasis than in those without metastasis (A and B). A total of 45 pairs of colorectal cancer tissues and adjacent tissues were collected. Quantitative polymerase chain reaction analysis of C. difficile abundance in cancer tissues revealed a significant increase compared to that in adjacent tissues, with even greater abundance in situ cancer tissues from patients with lymph node metastasis (C and D). Fluorescence in situ hybridization experiments detected C. difficile in the lymph node metastases of colorectal cancer patients (E and F). Coculture of colorectal cancer cells (HCT-116 and LoVo) with C. difficile significantly promoted the in vitro migration of cancer cells, as shown by Transwell migration assays (G and H) and scratch assays (I). In a mouse model of colorectal cancer lung metastasis established via tail vein injection, coculture of colorectal cancer cells significantly increased the number and size of metastatic foci in the lung (J and K). In a mouse model of colorectal cancer liver metastasis established via splenic injection, the coculture group showed a significantly increased number of liver metastatic foci (L).

Through correlation analysis of the 5 selected gut microbial markers with CRC groups and clinical staging, the crucial role of *Fn* in the occurrence and progression of CRC was deduced. To further support this finding, another set of samples was collected, which included fecal samples from CRC patients (n = 49) and healthy individuals (n = 30). Using qPCR, the abundance of *Fn* in fecal samples was measured, and a significant increase was found in abundance in the fecal samples of patients with CRC. Moreover, the fecal samples from patients with lymph node metastasis had significantly greater levels of *Fn* than those from patients without metastasis (Figure 4A and B).

Forty-five pairs of CRC tissue and adjacent normal tissue samples were also collected. Through qPCR, a significant increase was observed in the abundance of *Fn* in cancer tissue compared to that in adjacent normal tissue. Additionally, in situ hybridization experiments detected *Fn* in lymph node metastases in CRC patients (red dots indicated by arrows, Figure 4E and F). After coculturing CRC cells (HCT-116 and LoVo) with *Fn*, Transwell migration assays (Figure 4G and H) and scratch assays (Figure 4I) were conducted; the results revealed that coculturing with *Fn* significantly enhanced the in vitro migration of CRC cells. In a mouse model of CRC lung metastasis following tail vein injection, coculturing CRC cells significantly increased the number and size of lung metastatic foci (Figure 4J and K). In a mouse model of CRC liver metastasis following splenic injection, the coculture group showed a significant increase in the number of liver metastatic foci (Figure 4L). These collective findings substantiate the pivotal role of *Fn* in promoting CRC metastasis, in both clinical samples and experimental settings.

## Evaluation of Gut Microbial Markers as Diagnostic Biomarkers for Colorectal Cancer

Considering the results mentioned above, among the 5 biomarkers, Lactobacillus and Bifidobacterium exhibited no significant differences in their abundances across the normal group, the colorectal adenoma group, and the CRC group (Figure 1A and B). Conversely, *Fn*, *P*.* anaerobius*, and enterotoxigenic *Bacteroides fragilis* showed notable differences in their abundances across these groups, and they displayed a high correlation with CRC at different stages; these findings particularly highlighted the pivotal role of *Fn* in CRC metastasis (Figure 4). Therefore, these 3 gut microbial markers were selected for monitoring CRC. To explore their diagnostic value in CRC, ROC curves were generated to determine the cutoff values for distinguishing the CRC group from the non-CRC group (adenoma group and HC group).

**Figure 5.**
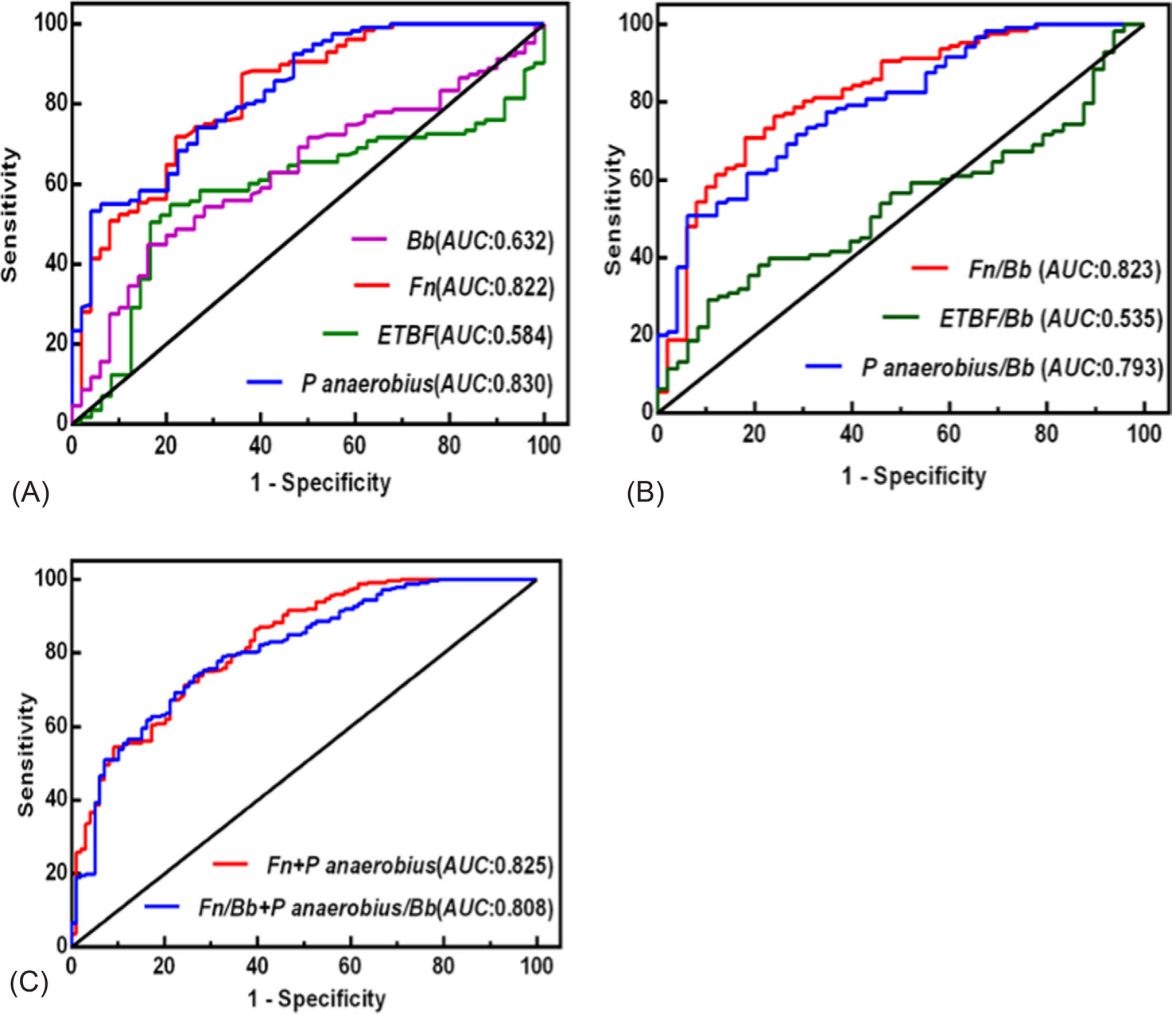
Receiver operating characteristic. Curve s of gut microbial markers for the diagnosis of colorectal cancer. The area under the receiver operating characteristic curve (AUC) for *Fn*, anaerobic Streptococcus, Bifidobacterium, and *Fn* toxin-producing fragile strains (A). The AUC for the ratio of *Fn*, *Fn* toxin-producing fragile strains, and anaerobic Streptococcus to Bifidobacterium (B). The AUC for *Fn*, anaerobic Streptococcus, and the combination of both compared to Bifidobacterium (C).

As shown in Figure 5, the AUCs for *Fn* and *P*.* anaerobius* were 0.822 and 0.830, respectively, while those for Bifidobacterium and enterotoxigenic *Bacteroides fragilis* were 0.632 and 0.584, respectively (Figure 5A). The AUCs for the ratios of *Fn*, enterotoxigenic *Bacteroides fragilis*, and *P*.* anaerobius* to Bifidobacterium were 0.823, 0.535, and 0.793, respectively (Figure 5B). When *Fn* and *P*.* anaerobius* were combined, or when their ratios with Bifidobacterium were combined, the AUCs were 0.825 and 0.808, respectively (Figure 5C). This result indicates that among the individual microbes, *P*.* anaerobius* had the highest diagnostic value for CRC (AUC: 0.830), followed by *Fn* (0.822). The combination with the highest diagnostic value was *P*.* anaerobius* in combination with *Fn* (0.825).

Regarding the comparisons between the HC and Colorectal Adenoma (CRA) groups, the effect sizes and 95% confidence intervals (CIs) for the microbial markers are as follows: for *Fn*, the Cohen’s d was −2.21, with a 95% CI of [−2.28, −1.66], indicating a large effect size in favor of the CRA group (AUC: 0.822). For *P*. *anaerobius*, the Cohen’s d was −2.21, with a 95% CI of [−2.28, −1.66], suggesting a large effect size (AUC: 0.830). For Bifidobacterium, the Cohen’s d was 0.23, with a 95% CI of [−0.35, 1.67], reflecting a small effect size (AUC: 0.632). For ETBF, the Cohen’s d was −2.21, with a 95% CI of [−2.28, −1.66], showing a large effect size (AUC: 0.584). These results indicate that among the individual microbes, *P*. *anaerobius* had the highest diagnostic value for CRC (AUC: 0.830), followed by *Fn* (AUC: 0.822). The combination with the highest diagnostic value was *P*. *anaerobius* in combination with *Fn* (AUC: 0.825), which showed the strongest diagnostic potential.

## Discussion

The gut microbiota, often referred to as the “second genome” in humans, perturbations in microbiota homeostasis due to external factors can precipitate a cascade of gastrointestinal responses, including stress, inflammation, immune responses, and neoplastic transformations. A burgeoning body of research implicates the gut microbiota in the pathogenesis and progression of CRC. Reports in the literature suggest that the human gut harbors an abundance of symbiotic bacteria, such as *Fn*, *Bacteroides fragilis*, and *Streptococcus bovis*, which are implicated in CRC development.^12^ These organisms not only affect a multitude of the biological behaviors of tumors—such as augmenting neoplastic cell proliferation—but also induce tumoral DNA damage and gene mutations, as well as promoting the formation of proinflammatory microenvironments and mediating tumor immune evasion.^13^^-^^15^ Notably, recent research discovered that the toxin released by ETBF stimulates the production of IL-17 in colon epithelial cells, which in turn initiates a mucosal immune response and promotes tumor-associated myeloid infiltration, thus exacerbating CRC development.^16^

At present, the conventional definition of groups with a “high-risk” of CRC—encompassing factors such as age, family history, and dietary patterns—lacks precision, which hinders the identification of individuals who need colonoscopic examination. Numerous investigations utilizing large-scale metagenomic cohort studies on CRC have identified 7 bacterial species that are notably enriched in CRC patients across 526 metagenomic samples from multiple countries, including *Bacteroides fragilis* and *Fusobacterium nucleatum (Fn)*. These species have demonstrated utility in distinguishing CRC patients from HC subjects.^17^ Previous studies have reported that a random forest classification model based on the fecal microbiota, in conjunction with the FOBT, substantially increases the diagnostic sensitivity for CRC.^18^^-^^20^ Also, the research has demonstrated that *Fn* enhances the migration of CRC cells in both cell and animal models, as well as in clinical samples. This suggests that *Fn* may be a pivotal factor in the progression and spread of the disease. The current landscape of CRC prognosis is challenged by a lack of robust and reliable biomarkers for different stages of the disease.^21^^,^^22^ This dysbiosis may contribute to alterations in the local and systemic inflammatory responses, potentially influencing tumor growth and metastasis. Understanding how *Fn* and other microbial factors interact with the tumor microenvironment could provide valuable insights into novel therapeutic approaches and improve the ability to predict and manage CRC.

However, several limitations of this study should be acknowledged. First, the observed associations between *Fn*, *P. anaerobius*, and CRC progression do not establish causation, especially given the cross-sectional design of the analysis, which provides no insight into temporal dynamics. Second, the detailed mechanistic pathways by which these microbes might contribute to tumor development and metastasis were not investigated, leaving the biological link between the gut microbiota and CRC progression unclear. Third, because all participants were recruited from a single center, the generalizability of the findings to other populations or regions is limited. Finally, one cannot rule out residual confounding from factors such as diet, lifestyle, and comorbidities that were not fully captured in the data. These findings suggest that alterations in gut microbiota composition may not only reflect the presence of CRC but also provide insights into disease progression and metastatic potential. Moreover, the observation of distinct expression patterns of microbial markers across CRC patients in different stages highlights the potential for gut microbiota monitoring as a noninvasive tool for CRC risk stratification and personalized treatment selection.^23^^-^^27^ This personalized approach to CRC management has the potential to optimize therapeutic efficacy and improve patient outcomes.

However, several important considerations warrant further exploration. First, the mechanisms underlying the observed associations between the gut microbiota and CRC remain incompletely understood. Future research efforts should focus on elucidating the biological pathways through which the gut microbiota influences CRC development and progression.^28^ Moreover, the clinical translation of microbial markers into routine practice necessitates rigorous validation in independent cohorts to confirm their diagnostic and prognostic utility across diverse patient populations.

In conclusion, the pivotal role of gut microbiota monitoring in CRC management was underscored. The identification of microbial markers associated with CRC staging and metastasis represents a significant advancement in gastroenterology research, with the potential to revolutionize CRC screening, diagnosis, and treatment. Continued research efforts are essential to unravel the complexities of gut microbiota-CRC interactions and translate these findings into tangible clinical benefits for patients.

## Figures and Tables

**Table 1.** The primer sequences for quantitive PCR

Target		Sequence	Company
*Fn*	Forward	CAACCATTACTTTAACTCTACCATGTT	TsingkeBiotechnologyCo., Ltd.
	Reverse	GTTGACTTTACAGAAGGAGATTATGT	
*P*. *anaerobius*	Forward	GGTGCGATGAAGAAGTGGTT	TsingkeBiotechnologyCo., Ltd.
	Reverse	GCAATCTTTGGGAGCATGTG	
ETBF	Forward	GGG ACAAGGATTCTA CCAGCTTTATA	TsingkeBiotechnologyCo., Ltd.
	Reverse	ATTCGGCAATCTCATTCATCATT	
Lb	Forward	AGCAGTAGGGAATCTTCCA	TsingkeBiotechnologyCo., Ltd.
	Reverse	CACCGCTACACATGGAG	
Bb	Forward	CTCCTGGAAACGGGTGG	TsingkeBiotechnologyCo., Ltd.
	Reverse	GGTGTTCTTCCCGATATCTACA	
Universal 16S	Forward	CGGCAACGAGCGCAACCC	TsingkeBiotechnologyCo., Ltd.
	Reverse	CCATTGTAGCACGTGTGTAGCC	

ETBF, enterotoxigenic Bacteroides fragilis; *Fn*, *Fusobacterium nucleatum*; *P*. *anaerobius, Peptostreptococcus anaerobius*; Lb, *Lactobacillus genus*; Bb, *Bifidobacterium genus*.

**Table 2.** Summary of Colorectal Cancer Fecal Microbiota Metagenomic Sequencing Data in Databases

Country	Dataset	Groups (n)	Microbial Signatures
France	ZellerG, 2014^2^	Control (61) Adenoma (42) CRC (53)	Twenty-two gut microbial species
China	YuJ, 2017^3^	Control (54) CRC (74)	*Fusobacterium nucleatum*, *Parvimonas micra* 20 gene markers
Austria	FengQ, 2015^4^	Control (61) Adenoma (47) CRC (46)	Two microbial community types
USA	VogtmannE, 2016^5^	Control (52) CRC (52)	Four gene markers
**Total**		**Control (** **228** **)** **Adenoma (** **89** **)** **CRC (225)**	

CRC, colorectal cancer.

**Table 3.** Summary of Microbial Biomarkers in Fecal Samples from Healthy Individuals, Adenoma Patients, and Patients with Colorectal Cancer in Databases

Disease (n)	Microbiome Signatures
Control (228)	*Bifidobacterium longum*, Ruminococcus, *Faecalibacterium prausnitzii* **,** *Bifidobacterium adolescentis*, *Lactobacillus fermentum*, *Bifidobacterium catenulatum*, *Eubacterium hallii*, *Bacteroides intestinalis*, and *Streptococcus salivarius*
CRC (225)	*Fn*, *Parvimonas micra*, *Peptostreptococcus stomatis*, *Peptostreptococcus anaerobius*, enterotoxigenic *Bacteroides fragilis*, *Prevotella stercorea*, *Escherichia coli*, *Gemella morbillorum*, *Solobacterium moorei*, *Clostridium symbiosum*, and *Anaerococcus obesiensis*
Adenoma (89)	*Collinsella aerofaciens*, *Staphylococcus aureus*, *Rothia dentocariosa*

CRC, colorectal cancer.

**Table 4.** Basic Characteristics of the Study Subjects (Mean ± SD)

	HC Group (n = 65)	CRA Group (n = 65)		CRC Group (n = 50)	*P*
		Non-AA (n = 40)	AA (n = 25)		
Age (years)	60.5 ± 5.7	60.1 ± 7.5		63.7 ± 8.4	>.05
Gender (M/F)	36/29	27/13	11/14	28/22	>.05
BMI (kg/m^2^)	24.0 ± 3.0	23.9 ± 3.6	23.7 ± 4.1	23.4 ± 3.0	>.05
Site of adenoma or CRC					>.05
Ascending	-	8	4	6	
Transverse	-	7	3	6	
Descending	-	2	5	5	
Sigmoid	-	10	12	13	
Rectum	-	6	8	20	

P values were calculated to assess the statistical significance of differences between groups.

AA, advanced adenoma; BMI, body mass index; CRA, colorectal adenoma; CRC, colorectal cancer; F, female; HC, healthy control; M, male.

## Data Availability

The data that support the findings of this study are available on request from the corresponding author.
